# The Great Failure of Malaria Control in Africa: A District Perspective from Burkina Faso

**DOI:** 10.1371/journal.pmed.0040127

**Published:** 2007-06-05

**Authors:** Bocar Kouyaté, Ali Sie, Maurice Yé, Manuela De Allegri, Olaf Müller

## Abstract

Too many African children are dying from a disease for which we have effective and cost-effective prevention and treatment options, say the authors.

Malaria remains the most important parasitic disease affecting humans [1]. Every year, there are some 5 billion clinical episodes resembling malaria, some 600 million clinical malaria cases, and about 1 million malaria deaths [2]. The great majority of the malaria burden falls on the poor rural communities in sub-Saharan Africa (SSA), and most deaths occur in young children [1,2]. Malaria is considered a major barrier to the development of SSA [3].

The rapid spread of resistance against chloroquine, for decades the most important safe, effective, and affordable antimalarial drug worldwide, was considered a public health disaster for SSA by 1998 [4]. One year later, a group of leading malariologists called for the systematic employment of combination therapy, preferably artemisinin-based combination therapy (ACT), to avert the further spread of drug resistance and thus a global malaria catastrophe [5]. In the meantime, the grave public health consequences of chloroquine resistance in terms of increasing childhood mortality in SSA have been well described [6,7]. As a consequence, 23 countries in SSA had changed their first-line malaria treatment policy to ACT by the end of 2004, and many more have followed up to the present [8]. However, because of its cost and short supply, ACT remains first-line therapy in policy but not in practice [1,9,10].

In the field of malaria prevention, insecticide-treated mosquito nets (ITNs) have been developed as a promising tool over the last two decades [1]. ITN protection is roughly associated with a 50% reduction of malaria morbidity and a 20% reduction of all-cause mortality in children, and these effects are sustained over time [11,12]. However, 25 years after the first report on the benefit of this intervention, ITN coverage in young children is still unacceptably low in SSA [13].

Here we present an analysis of the malaria situation in one typical district of Burkina Faso, which likely represents the pattern of problems seen in many SSA countries today.

## Health Services and Research Capacity

Burkina Faso, situated in the Sahel zone of West Africa, is one of the poorest countries in the world [14]. State and external aid cover respectively 18% and 28% of all health expenditure; the remaining 54% is financed directly by the population [14]. Governmental health spending equals US$9 per capita per year. A total of three teaching hospitals, 11 regional hospitals, and 55 district hospitals serve the country. While the regional hospitals have specialist units and are run by physicians, the district hospitals are run by nurses with some supervision, usually from two physicians in charge of the whole district. Consequently, formal health services for the rural population are limited to small health centres staffed by two nurses and one midwife.

The National Malaria Control Programme (NMCP) is a rather small Ministry of Health unit (three physicians, seven other staff). In contrast, four research centres in the country are engaged in malaria research: The Centre National de Recherche et de Formation sur le Paludisme and the Institut de Recherche en Sciences de la Santé in the capital city Ouagadougou, the Centre Muraz in Bobo Dioulasso, and the Centre de Recherche en Santé de Nouna in Nouna (Figures 1 and 2). These centres receive various external support ranging from academic collaborations to longstanding capacity-building grants from industrialised countries. Overall, some 50 scientists are working on malaria in these institutions.

**Figure 1:**
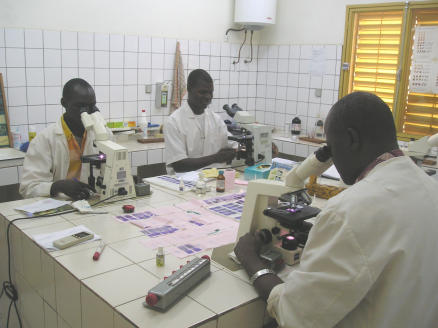
Lab Technicians Reading Slides from a Malaria Drug Trial at the Nouna Health Research Centre (Photo: Olaf Müller)

**Figure 2:**
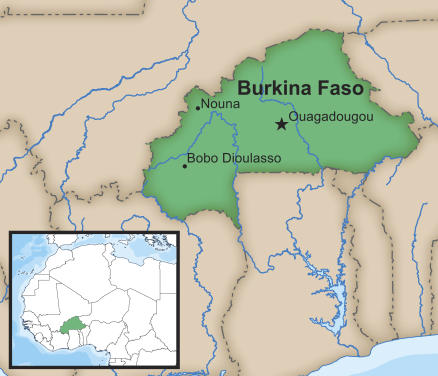
Location of the Four Malaria Research Centres in Burkina Faso The four centres are the Centre National de Recherche et de Formation sur le Paludisme and the Institut de Recherche en Sciences de la Santé in the capital city Ouagadougou, the Centre Muraz in Bobo Dioulasso, and the Centre de Recherche en Santé de Nouna in Nouna. (Figure: Anthony Flores)

## Malaria in Burkina Faso

Malaria in Burkina Faso, predominantly caused by Plasmodium falciparum, is highly endemic and the leading cause for morbidity and mortality [15,16]. In vivo chloroquine resistance was first reported in 1988 and clinical failure rates in children with uncomplicated malaria were around 5% in the early 1990s [17]. However, more recent data have shown a rapid increase of chloroquine but not pyrimethamine–sulfadoxine resistance over the last few years [18–21].

In February 2005, the Ministry of Health called for a national expert meeting to discuss alternative malaria treatment options. It was decided to switch from chloroquine to ACT and this information was circulated to all governmental health workers by April 2005 [22]. In a further correspondence to the formal health services, this policy change was reinforced by the Ministry of Health in June 2006 despite mention in the same document that ACT was still not available through governmental drug channels [23].

## Malaria in the Nouna Health District

Nouna Health District (NHD) is situated in north-western Burkina Faso. It has a population of 296,000 living in 274 villages served by 25 local health centres and by a district hospital located in the provincial capital, Nouna (population 25,000).

Malaria is holoendemic but highly seasonal in NHD, with most cases occurring during or briefly after the rainy season, which lasts from June until October [15]. Most of the malaria burden is in children, with preschool children experiencing at least six fever episodes and two malaria episodes per year [16]. Malaria is the major cause for the childhood mortality rate of 35 per 1,000 per year [24,25]. Chloroquine treatment of preschool children in 2001 failed in 10% on day 14, but failure rates subsequently increased significantly ([26] and B. Kouyaté, unpublished data). In 2003, this figure was above 50% in Nouna town [21].

Malaria control is based on home treatment of fever cases with a mix of antipyretics, chloroquine, and traditional treatments [27]. Only a minority of patients are treated in formal health services [27], and many antimalarials are substandard drugs bought at markets. The great majority of children who die of malaria had not visited formal health services during their final illness [27].

On average and considering exclusively direct costs, households in NHD spend about US$2 to treat one fever episode [28]. Considering that a child suffers about six episodes of fever per year, an average household with four children may end up spending US$48 just for presumptive malaria treatment in children.

In spite of the official policy change, ACT continues to be available only in private pharmacies in the town of Nouna and at a prohibitively high price (US$6.5 for the course of treatment needed for a child). A study conducted in early 2006 on a representative sample of 1,080 NHD households demonstrated that of 122 fever episodes in preschool children, none was treated with ACT, 112 were treated with chloroquine, six with amodiaquine, three with quinine, and one with pyrimethamine–sulfadoxine (M. Tipke, unpublished data). This treatment behaviour may also be influenced by the experience of chloroquine being a good anti-inflammatory drug in cases of febrile illness. However, even governmental health workers in Nouna town are still prescribing chloroquine for the treatment of fever in young children.

ITN provision in the NHD was until recently limited to project activities at the Centre de Recherche en Santé de Nouna and to small-scale sales supported by social marketing in Nouna town [12]. However, it has been shown that demand for ITNs is high and that compliance with freely distributed ITNs under the conditions of an effectiveness trial was good [29,30].

## Discussion

The difficulties of translating research findings into practice are well known and have been found to be a universal phenomenon [31]. With regard to evidence-based changes of malaria treatment policies, such processes have already been described in detail for selected SSA countries [32,33].

The development of clinically relevant resistance against chloroquine in Burkina Faso was discovered through studies conducted by national health research centres. This was followed by a national meeting of experts and—forced by strong recommendations from the World Health Organization (WHO)—to a policy shift to ACT as recommended treatment. However, for the last two years this policy has been only on paper as ACT is not available through governmental health services. The obvious reason for this is lack of funds. The Global Fund to Fight AIDS, Tuberculosis and Malaria (GFATM) is the main external funding body for ACT in SSA [1,34]. However, a proposal to the GFATM on malaria control support in Burkina Faso was rejected in June 2005. This decision is widely perceived in Burkina Faso as mainly motivated by financial problems of the GFATM.

Another obvious fact is that malaria research capacity appears to be well developed in Burkina Faso. However, the implementation capacity of the NMCP is not. As we are aware of similar mismatches between research and implementation capacities in other SSA countries, we may question whether the tendency of external donors and malaria-endemic countries to invest much into malaria research, while at the same time neglecting support for capacity building and strengthening of national programmes, is justified.

So what would an ideal malaria control programme look like in a country such as Burkina Faso? First of all, safe and effective drugs need to be available and accessible to the whole population. High treatment coverage with well-equipped health services is unlikely to be implemented in the near future, particularly in the rural parts of the country. One approach to overcome this obstacle could be a programme of home- and community-based malaria treatment, and promising experience on this topic has been demonstrated (B. Kouyaté, unpublished data), [35]. However, the choice of drugs is crucial and the cost could be prohibitive: if all the roughly 20 million annual fever episodes occurring in preschool children in Burkina Faso were treated with ACT at a cost of US$1 per course, this would already amount to US$20 million [2]. Moreover, protection of all preschool children with long-lasting ITNs would add another US$20 million as initial investment costs followed by US$5 million per year for replacements [2,36].

Nevertheless, policy makers must realise that malaria prevention and treatment interventions clearly belong together, as ITN protection for example would roughly halve the number of malaria episodes and consequently ACT treatment courses [11]. An estimated US$2–3 billion of external funds would be needed every year to scale up the response against malaria in all of SSA, but investments are presently only in the range of US$100 to 200 million [37]. The amount that the impoverished populations of SSA could contribute towards these investments is negligible [36,37].

Unfortunately there is no ideal world. As sufficient funds for high coverage provision of ACT are currently not available, an appropriate interim solution would be to use a pragmatic combination of two affordable drugs. The obvious choice would be the combination of pyrimethamine–sulfadoxine and amodiaquine, which has been shown to be as effective as ACT in a number of SSA countries, including Burkina Faso [38–40].

However, after it became clear that Burkina Faso would not receive GFATM funds for the purchase of ACT, the NMCP of Burkina Faso asked the World Bank to use a portion of an existing US$12 million loan from the Global Strategy and Booster Program to purchase pyrimethamine–sulfadoxine and amodiaquine as an interim solution. This request was rejected with the argument that WHO recommends only ACT. As a result, chloroquine remains factually the first-line malaria treatment in Burkina Faso. These observations support the view that SSA countries continue to be victims of ignorance and lack of coordination between external donors and international organisations [41,42].

In summary, we call for more realistic malaria control policies and their rapid and comprehensive implementation in SSA. Too many African children are dying in these days from a disease against which effective and cost-effective prevention and treatment options have long been developed.

## Abbreviations

ACT: artemisinin-based combination therapy
GFATM: Global Fund to Fight AIDS, Tuberculosis and Malaria
ITN: insecticide-treated mosquito net
NHD: Nouna Health District
NMCP: National Malaria Control Programme
SSA: sub-Saharan Africa
WHO: World Health Organization
